# Keep calm and transcribe on: chromatin changes with age, but transcription can learn to live with it

**DOI:** 10.15252/msb.202211276

**Published:** 2022-09-14

**Authors:** Cian J Lynch, Manuel Serrano

**Affiliations:** ^1^ Cellular Plasticity and Disease Group, Institute for Research in Biomedicine (IRB Barcelona) Barcelona Institute of Science and Technology (BIST) Barcelona Spain; ^2^ Catalan Institution for Research and Advanced Studies (ICREA) Barcelona Spain

**Keywords:** Chromatin, Transcription & Genomics

## Abstract

Assessing age‐related tissue dysfunction represents an emerging field and involves analyses that are far from trivial, often requiring the integration of several large‐scale (“omic”) techniques. In their recent work, Tessarz and colleagues (Bozukova *et al*, 2022) characterize changes in the transcriptional machinery during aging in mice and report some surprising findings.

## Graphical abstract

Integrating different genome‐wide data can be powerful in assessing age‐related tissue dysfunction. In their recent study, Tessarz and colleagues (Bozukova *et al*, 2022) report increased chromatin accessibility in aged mouse liver, which is however not accompanied by increased transcriptional output.

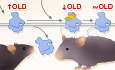

Tissue function declines with age—perhaps one of the earliest and simplest biological observations. But over the last two decades, technological advances and in‐depth analyses have triggered the realization that aging is much like any other biological process: amenable to mechanistic understanding and perhaps even manipulation. At the molecular level, aging manifests as decreased coherence in our biological clockwork (López‐Otín *et al*, 2013). Moreover, an ever‐growing list of age‐associated alterations in signaling pathways and gene expression continues to be characterized (Tabula Muris Consortium, 2020). An important nexus of all these inputs and outputs is the transcriptional machinery itself.

A complex sequence of events precedes successful gene transcription. In brief, chromatin is first activated and opened, rendering it receptive to transcription. This is followed by the recruitment and assembly of RNA Pol II on the target gene and, lastly, RNA Pol II is released into productive elongation, a process that is intricately regulated. In a tour de force, Bozukova *et al* assess each of these stages for all transcribed genes, in liver tissue from young and old mice (Bozukova *et al*, 2022). They then integrate the genome‐wide information, assembling a comprehensive overview of age‐associated changes. Altogether, this constitutes the first systematic whole organ study that combines chromatin accessibility, analysis of core transcriptional machinery, and transcriptomic output, per gene, and for all genes.

As a summary readout of chromatin activation status, the authors first quantified chromatin accessibility across the genome, using the well‐established technique ATAC‐seq. Interestingly, the old liver displayed a global increase in accessibility to gene promoters and enhancers. Typically, more open chromatin is associated with an increased potential in target gene expression. However, the gene expression differences in the liver of young vs old mice were moderate and did not correlate with the global increase in chromatin accessibility at promoters. This counterintuitive observation suggested that the function of RNA Pol II transcriptional machinery may change during aging. Thus, Bozukova *et al* next performed a detailed analysis of RNA Pol II function.

Compared with histones and most chromatin regulators, RNA Pol II is highly dynamic and moves along genes. Therefore, an experimental snapshot of the distribution of RNA Pol II along a gene is complex and reveals underlying checkpoints in the control of transcriptional elongation (Roeder, 2019; Schier & Taatjes, 2020). Notably, RNA Pol II assembles at the promoter start of transcription (around position +1 bp), and typically arrests transiently during early elongation at a “pause site” (around positions +20 to +60 bp) where control mechanisms can eject the complex or license it for full elongation (Chen *et al*, 2018). Bozukova *et al* used a combination of techniques to compare RNA Pol II function in old vs young mouse liver and to precisely map the abundance of RNA Pol II with single base‐pair resolution along every gene. Crucially, they observed a striking and global reduction in paused RNA Pol II in the old liver samples. To explore this, the authors examined some known regulators of the pausing process. Of note, the accessory factor SPT4, which is important for licensing into elongation, was depleted from promoters across the genome in old livers. This provides a potential explanation for the apparent paradox whereby aging is associated with more accessible chromatin at promoters, without the expected increase in transcriptional output. The authors conclude that the increased chromatin accessibility to RNA Pol II is compensated by a reduced efficiency in elongation. Specifically, the potential increase in RNA Pol II recruitment and initiation is offset by a destabilized pausing complex and ejection of RNA Pol II from the gene. Ultimately, it is tempting to speculate that a compensation process is at work, where increased chromatin accessibility is mechanistically linked to a deficient promoter pausing of RNA Pol II and reduced engagement into full elongation, thereby preserving normal gene expression levels (Fig 1).

**Figure 1:**
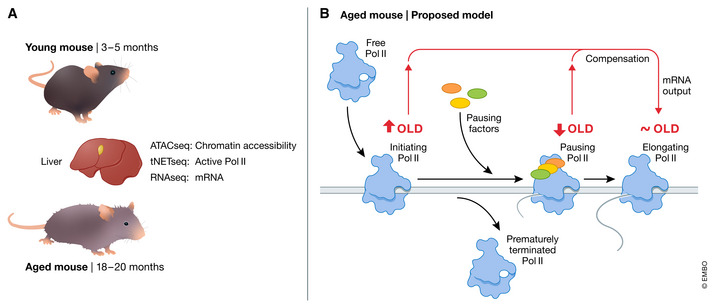
Age‐related changes in transcription in the mouse liver (A) Integrative genome‐wide analyses of chromatin accessibility and transcription in young vs. old mouse liver samples indicated increased chromatin accessibility in old animals, which is, however, not accompanied by the expected increase in transcriptional output. (B) The model proposed by Bozukova *et al* suggests that increased chromatin accessibility to RNA Pol II is compensated by a reduced efficiency in elongation due to deficient promoter pausing of RNA Pol II and ultimately premature termination of transcription.

This study raises fundamental questions that remain to be further explored: how does chromatin accessibility increase, while RNA Pol II pausing decreases, with age? Are these processes causally linked, or could they be independently generated phenomena? If they are linked, which process is the initiator? Future studies can continue to probe this mechanistically since the pausing process has been previously studied in detail, and many more regulators are known (Chen *et al*, 2018; Schier & Taatjes, 2020). For analyzing regulators and global trends in pausing and chromatin accessibility, future studies may routinely include spike‐ins to monitor for global changes in transcription output. Notably, a recent analysis of transcription at the single‐cell level during aging has revealed a global reduction in RNA levels, including hepatocytes (Pálovics *et al*, 2022). Other levels of complexity can emerge upon aging such as transcriptional dispersion or heterogeneity between cells of the same type (Nikopoulou *et al*, 2019). This could also include cell‐to‐cell heterogeneity in chromatin accessibility and RNA Pol II pausing. It remains to be determined if the transcriptional compensation model, described by Bozukova *et al* in bulk tissue, comes at the price of increased cell‐to‐cell heterogeneity. In summary, the current work puts dysregulation of chromatin accessibility and transcriptional pausing in the focus of aging research.

## Disclosure and competing interests statement

MS is a shareholder of Senolytic Therapeutics, Life Biosciences, Rejuveron Senescence Therapeutics and Altos Labs, and is advisor of Rejuveron Senescence Therapeutics and Altos Labs. The funders had no role in the study design, data collection and analysis, decision to publish, or manuscript preparation.
